# Stopping attacks on health care

**DOI:** 10.2471/BLT.22.020822

**Published:** 2022-08-01

## Abstract

Improving the reporting of attacks on health care is only part of the solution to reducing their occurrence. Tatum Anderson reports.

Dr Houssam al-Nahhas’s first experience of an attack on health care was an attack on himself. “It happened in Aleppo in August 2012,” he says. “I was a medical student at the time and had been coordinating a clandestine health network helping to treat people who had been injured in demonstrations against the Syrian government.”

Detained by the Syrian police for 16 days, al-Nahhas was interrogated and tortured. “The entire interrogation was focused on my work as a health-care provider,” he says. “They wanted to know who I was treating, how I got my supplies, and who was working with me.”

Ten years later, al-Nahhas works as a Middle East and North Africa investigative researcher with Physicians for Human Rights, a nongovernmental organization (NGO) that documents severe human rights violations and atrocities around the world to support advocacy and accountability.

The issue has received increased attention in recent years, notably since the launch of the World Health Organization’s (WHO) Attacks on Health Care initiative in 2015, followed by a United Nations (UN) Security Council resolution (2286) condemning such attacks which was passed in May 2016.

Co-sponsored by 80 countries, a key aim of the resolution was to reaffirm the protections provided under international humanitarian law enshrined in the Geneva Convention, the 1949 revision of which explicitly references protection for medical and religious personnel, medical units and medical transports.

Despite these developments, progress has been slow. For Hyo-Jeong Kim – who leads WHO’s Surveillance System for Attacks on Health Care (SSA), one of the primary outputs of the Attacks on Health Care initiative – accurate monitoring and documentation is vital to making progress on the issue.

“Monitoring is key to establishing the evidence base we need to better understand attacks on health care and develop policies to combat them,” she says.

To reflect the full complexity and extent of the problem, SSA throws the surveillance net wide, using 15 categories of attack, ranging from assaults with heavy weaponry to acts of violence or obstruction, whether psychological or physical, that prevent access to health care.

Reports of alleged attacks are collected by WHO country offices through email, text messaging, an online system and/or dedicated app, and are corroborated by country information officers using other sources of information including, where possible, first-person testimonies or reports from trusted NGOs on the ground. Once verified, each report is classified according to four levels of certainty ranging from “rumour” to “confirmed”.

“The challenge we face is how to encourage optimal reporting.”

Hyo-Jeong Kim

According to the SSA, reports of confirmed attacks on health care have increased in recent years, rising from 343 in 2020 to 832 confirmed attacks in 2021. As of 29 June 2022, 490 confirmed attacks had been reported this year, including 280 attacks with heavy weapons such as missiles, bombs and artillery shells. As of the same date, 339 of those attacks had been reported in Ukraine.

SSA does not report the contextual information it gathers such as the names and locations of people or places attacked, a policy that has sometimes attracted criticism from commentators understandably keen to hold perpetrators to account.

“The challenge we face is how to encourage optimal reporting which depends on informants feeling that they will not face retribution,” Kim explains. “Keeping their identity and location hidden is therefore vital.”

In some cases, fear of retribution has led to silence in situations where attacks were known to have taken place. This is true of Tigray in Ethiopia for example, where, according to the Ethiopian health ministry, in 2021 more than 1500 health facilities were destroyed or damaged in Amhara and Afar in the ongoing conflict between the Tigray People’s Liberation Front and the federal government. None of the attacks made it into the SSA.

And retribution is just one of the risks faced when too much information is shared. “A UN-facilitated deconfliction mechanism set up in Syria to reduce the risk of damage to health facilities involved sharing their location in the conflict zone,” recounts al-Nahhas. “It was found that after sharing all these GPS locations, facilities were more likely to be targeted by the Syrian government and its Russian allies.”

In some ways the flow of information regarding attacks on health care has never been richer, boosted by near-ubiquitous smart phones in most parts of the world. Users record and share images on social networks, while news agencies sometimes acquire images that have gone “viral” for dissemination on their broadcasting platforms.

However, because digital information is subject to manipulation, it is easy for perpetrators to dismiss reporting of their crimes as fake news. Bellingcat, a Netherlands-based investigative journalism group, specializes in fact-checking and digital investigation using publicly available data from satellite imagery to social media posts.

According to Nick Waters, who leads a Bellingcat team looking specifically at health care in war, footage surfaced in 2019 suggesting a missile caused an explosion in Beirut. Using photos of the explosion, the team was able to show that footage had been re-purposed from elsewhere. “We are very, very good at looking at a picture or video and working out exactly where it was filmed,” he says.

The capacity to execute multiple corroborations using sources that include satellite imagery also makes Bellingcat good at authenticating events, including disputed events such as the deliberate targeting of health facilities.

“In one case it became evident by comparing satellite images and video footage that one health-care facility was struck multiple times by precision-guided munitions,” Waters says, referencing the Kafr Nabl Surgical Hospital in the town of Kafr Nabl in southern Idlib, Syrian Arab Republic, which has been struck on numerous occasions, the last time in June 2019.

Bellingcat is currently focusing its digital monitoring efforts on the war in Ukraine. Whether such efforts eventually lead to prosecutions for attacks on health care, which constitute breaches of international humanitarian law, remains to be seen. However, Waters is hopeful that, knowing that their acts may be observed and recorded, would-be assailants may think twice about launching them.

“Prosecution of human rights violations [is needed].”

Houssam al-Nahhas

Maciej Polkowski, head of the Health Care in Danger Initiative at the International Committee of the Red Cross (ICRC), also believes that attack prevention is a realistic goal but focuses on measures other than monitoring and documentation.

The ICRC has developed a multi-layered strategy that encourages a range of actions from high-level ministerial actions to concrete, readily implementable strategies that can be used to prevent attacks occurring during conflicts.

These include making hospitals less vulnerable to the formation of armed mobs in conflict zones. “When there is a mass casualty incident or the prominent leader of the local community is killed, you frequently see hundreds of people crowding into emergency departments demanding faster provision of health care while at the same time effectively preventing it through their physical presence,” Polkowski says. To avoid this happening the ICRC has introduced screening processes within hospital foyers using security guards or metal detectors, so that weapons remain outside.

The ICRC also uses learning materials to educate doctors not to refuse treatment to enemy combatants, and de-escalation techniques to help health workers prevent tense situations escalating into violence. Teaching is even targeted at military personnel and requires ICRC workers to meet with military leaders to explain that demanding help for their own soldiers at the expense of other patients constitutes a violation of international humanitarian law.

Community involvement can also play an important role in preventing attacks against health care. This has been amply demonstrated in Borno, Adamawa and Yobe states in north-eastern Nigeria where Boko Haram and Islamic State West Africa have killed and abducted health workers and raided and destroyed hundreds of facilities since the uprising began in 2009. This has led to the departure of terrorized health-care workers across the insurgent-affected region.

“Even me and my teams were attacked in some areas,” says Saratu Ayuba, a WHO coordinator responsible for mobile health-care teams in the states.

To protect the teams, local communities have put together a Civilian Joint Taskforce, the members of which know the local landscape and local languages and can identify Boko Haram members. “They often send alerts via SMS to tell my teams not to come if the situation becomes too dangerous,” Ayuba says.

Local health-care professionals have also started reducing large drug deliveries, because such vehicles are more likely to be attacked. According to Dr Hassan Bala, a WHO public health officer based in Maiduguri, Nigeria, areas that were completely inaccessible for health services are opening up. “Highways that used to see many attacks have seen reductions,” he says.

Needless to say, prevention is of less value in situations where one state attacks the health system of another. For Physicians for Human Rights’ al-Nahhas, in such instances, ensuring accountability through effective documentation and prosecution under international law is the only response likely to have an impact.

“The truth is attacks on health-care systems are rarely prosecuted by an international mechanism,” he says. “It’s good that we are now hearing that the attacks in Ukraine are being investigated. These efforts should continue and should proceed further toward actual investigation into and prosecution of human rights violations. That’s really the only way we can push back.”

**Figure:**
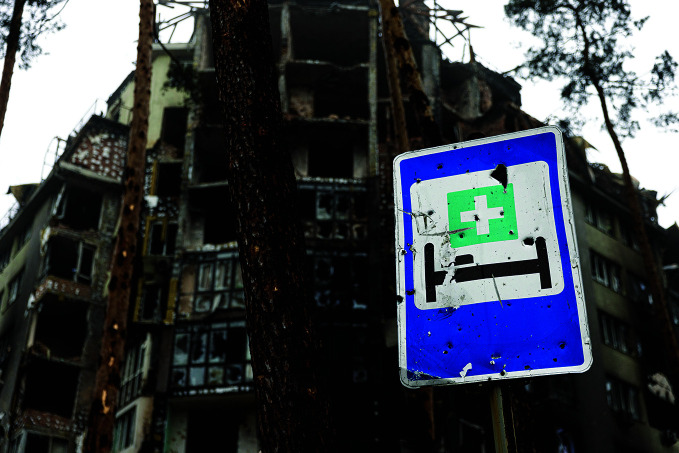
Destroyed military hospital in the city of Irpin, Ukraine.

ICRC/Alyona Synenko

**Figure:**
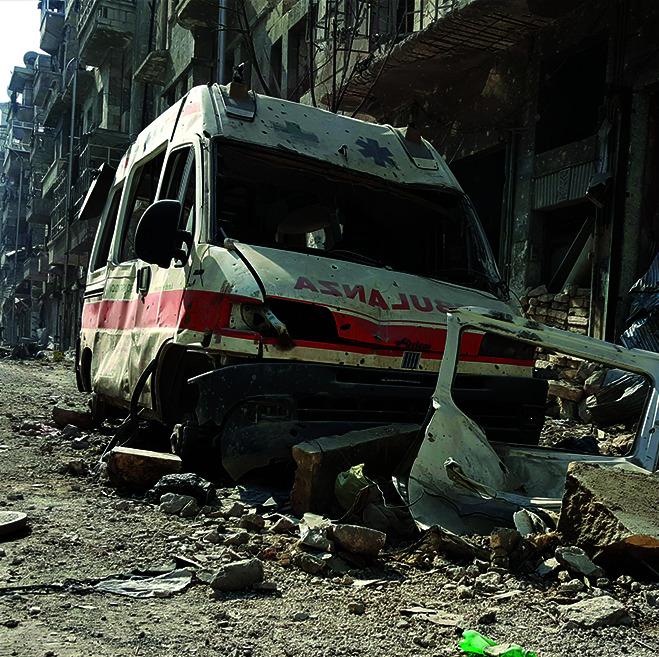
Destroyed ambulance in the Al-Kallaseh district of Aleppo, Syrian Arab Republic.

ICRC/Sana Tarabishi

